# Nonsurgical Management of Furcation Defects Using Cervical Sealing With Calcium–Silicate Cements: A Clinical Case Series

**DOI:** 10.1155/carm/8048506

**Published:** 2025-05-20

**Authors:** Saeed Asgary

**Affiliations:** Iranian Center for Endodontic Research, Research Institute for Dental Sciences, Shahid Beheshti University of Medical Sciences, Tehran, Iran

**Keywords:** calcium derivative, CEM, cervical seal, endodontics, furcation defects, mineral trioxide aggregate, minimally invasive

## Abstract

Furcation defects pose significant challenges in endodontic and periodontal therapy due to their complex anatomy and limited accessibility. Achieving a reliable seal at the apical/cervical/coronal levels is critical for long-term treatment success. This case series investigates the use of calcium–silicate biomaterials, specifically calcium-enriched mixture (CEM) cement, as cervical sealants in the nonsurgical management of furcation defects with endodontic origin, evaluating their regenerative potential and clinical applicability. Six endodontically treated teeth with furcation defects were included. All cases had undergone orthograde root canal therapy in the past and then were nonsurgically retreated with CEM cement placed as a cervical seal for this report. Baseline and follow-up evaluations, conducted over an average period of 31 months, assessed the clinical parameters of probing depths, furcation involvement, and radiographic evidence of healing. Radiographically, five cases demonstrated complete healing/regeneration, and one case showed partial resolution of the furcal lesion. Improvements in periodontal parameters, including lesser probing depths and elimination of bleeding and discharge, were observed across all cases, resulting in restored functionality of the affected teeth. The results suggest that CEM cement was an effective cervical sealing biomaterial for the nonsurgical management of furcation defects with endodontic origin. These findings highlight the potential of bioactive endodontic materials in minimally invasive dental therapies. Further studies with larger sample sizes and long-term follow-ups are needed to validate these findings.

## 1. Introduction

Furcation defects originate from infected accessory/chamber/furcation canal(s), and they represent a significant challenge in endodontic/periodontal therapy due to their anatomical complexity and the difficulty in achieving complete resolution through nonsurgical means [1]. These retrograde periodontal diseases are characterized by the loss of bone and attachment in the furcation area of multirooted teeth and often indicate a primary endodontic lesion with drainage through the periodontal ligament or secondary periodontal involvement [2]. Such lesions compromise the prognosis of affected teeth and pose difficulties in management, particularly in achieving an adequate seal to prevent reinfection and promote healing. The apical, cervical, and coronal seals are critical components for the long-term success of endodontic treatment [3], particularly in cases where furcation defects originate from endodontic pathologies via patent furcation canals [4].

Recent advances in endodontic biomaterials have introduced bioactive cements with unique properties, including biocompatibility, bioactivity, and the ability to stimulate hard tissue formation [5]. The calcium silicate cement-based products, such as mineral trioxide aggregate (MTA) named products, calcium-enriched mixture (CEM) cement, and other calcium silicate cement products, have demonstrated promising outcomes in various endodontic applications [6–8]. CEM cement exhibits favorable sealing abilities, antibacterial properties, and the potential to enhance periodontal regeneration, making it suitable for use in challenging cases like furcation defects of endodontic origin.

Surgical approaches have traditionally been the gold standard for managing furcation defects [9, 10] but have limitations of higher costs, higher patient discomfort, and the risk of further complications. Nonsurgical approaches, using bioactive materials, offer a minimally invasive alternative that may achieve similar clinical outcomes while preserving patient comfort and reducing treatment complexity.

This case series aims to evaluate the clinical efficacy of using CEM cement as a cervical seal in the nonsurgical management of furcation defects of endodontic origin. This clinical study assessed the regenerative potential, periodontal outcomes, and overall applicability of CEM cement in the nonsurgical management of furcation defects of endodontic origin. This work contributes to the growing body of evidence supporting the use of bioactive materials in minimally invasive therapies for periodontal lesions with an endodontic etiology.

## 2. Case Presentation

This case series included six mandibular molars from six patients (three females and three males) with a mean age of 39.83 ± 8.94 years (age range: 29–52). All patients had a history of previous endodontic treatment and furcation involvement. The primary complaints included local abscess formation, pus discharge, and dull pain in the furcal region. The patients had been referred to an endodontic private clinic, where systemic examinations were unremarkable, revealing no relevant medical histories or ongoing medications.

All cases had periodontal pockets exceeding 5 mm and Class II furcation involvement, accompanied by active pus discharge. Radiographic assessments showed that all teeth had undergone prior root canal therapy with root canal fillings in place. However, the radiographs highlighted the presence of furcal lesions, with the main issue identified as the absence of an adequate hermetic seal in the coronal and furcation areas of the root canal system. Based on these findings, the diagnosis of symptomatic furcal periodontitis of endodontic origin was established.

The inclusion criteria for this case series were as follows:

• Multirooted molars with furcation defects of endodontic origin.
• Radiographic evidence of furcation lesions with corresponding clinical signs and symptoms.
• Teeth suitable for salvage without extraction.
• Patient consent for nonsurgical treatment and follow-up evaluations.

Cases with vertical root fractures, systemic contraindications for treatment, or furcation defects unrelated to endodontic pathology were excluded.

The treatment plan focused on nonsurgical endodontic management using CEM (BioniqueDent, Tehran, Iran) cement as a cervical seal. The risks and benefits of the proposed treatment were thoroughly explained, and written informed consent was obtained from all patients.

**Figure 1:**
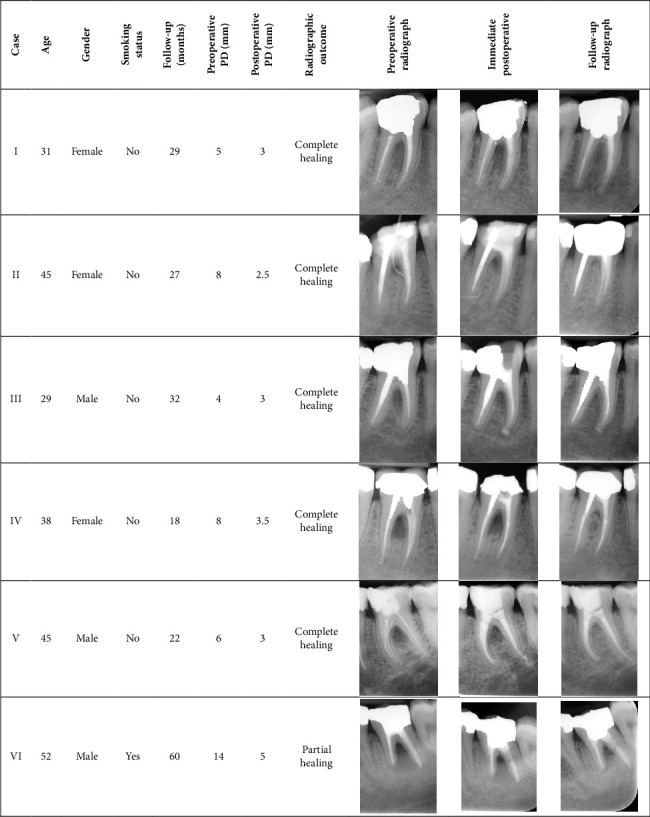
Patient characteristics: preoperative and postoperative outcomes.

The treatment procedures were performed by an experienced endodontist (Saeed Asgary) under local anesthesia using 2% lidocaine with 1:100,000 epinephrine (DarouPakhsh, Tehran, Iran). Access cavities were prepared using high-speed diamond burs. In cases where all canals had poor obturation, complete endodontic retreatment was performed (Figure 1; Case I). For teeth with acceptable obturation in some canals but poor obturation in others, only the poorly obturated canals were endodontically retreated (Figure 1; Case II-III). If prior root canal treatment was deemed acceptable in all canals, retreatment was not performed; instead, the coronal portions of the root canal fillings were removed (Figure 1; Case IV–VI). Chemomechanical preparation using the step-back technique with stainless steel K-files (Mani, Inc., Tochigi, Japan) for retreatment, accompanied by irrigation with 5.25% sodium hypochlorite and final irrigation with normal saline solution. The canals were then obturated using gutta-percha and sealer (Healapex, BioniqueDent, Tehran, Iran) via the lateral condensation technique. Where retreatment was not required, the prepared cavities were irrigated with 5.25% sodium hypochlorite and normal saline solution. CEM cement was mixed according to the manufacturer's instructions with approximately a 1/3 water/powder ratio and then all cases were sealed with incrementally placed and packed CEM cement. Coronal restorations were performed postendodontically, with the possibility of supplemental periodontal interventions if the healing process proved unsatisfactory.

The success of the treatment was assessed with clinical and radiographic evaluations annually. Clinical signs/symptoms subsided within 1 week, and all patients reported the resolution of pain and discomfort. Regular follow-ups confirmed significant improvements in periodontal parameters, including reduced probing depths (∼3 mm) and elimination of bleeding on probing.

Radiographic examinations (mean duration: 31 months and range: 18–60 months) showed complete resolution of furcal lesions in five cases (Figure 1; Cases I–V) and partial healing in one case (Figure 1; Case VI), even after 60 months. All treated teeth regained full functionality and remained symptom free throughout the long-term follow-ups.

## 3. Discussion

Furcation defects are often linked to complex endodontic and periodontal challenges and pose significant risks to the survival of multirooted teeth due to bone and attachment loss in the furcation area [11]. Traditionally, grade II furcation defects of endodontic origin have been managed through a combination of surgical and nonsurgical approaches [12]. Nonsurgical management offers a less invasive alternative while preserving tooth structure with the use of bioactive materials, such as calcium silicate cement-based products. Among these biomaterials, CEM cement has been shown to have excellent biocompatibility, bioactivity, and ability to promote hard tissue formation [13, 14]. CEM has favorable sealing properties, antibacterial effects, and potential to support periodontal regeneration [14]; therefore, it was an effective cervical seal in this study of nonsurgical management of furcation defects. The resolution of clinical symptoms—pus discharge, pain, and bleeding on probing—underscores the effectiveness of CEM cement in enhancing periodontal health in endodontic–periodontal lesions. All patients experienced complete subsidence of signs/symptoms and reported the restoration of normal chewing function, reflecting the ability of CEM cement to facilitate healing without the need for invasive procedures. Normal (< 5 mm) probing depths and the absence of active bleeding during the follow-up visits indicate that the periodontal health of all affected teeth was successfully restored. Five out of six cases showed complete resolution of furcal lesions, while one case—involving the oldest patient (52 years old), who was a smoker—exhibited partial healing. This suggests that smoking and advanced age may have contributed to the incomplete healing, underscoring the importance of patient-specific factors in regenerative outcomes [15]. Despite this, the overall results highlight the regenerative potential of CEM cement in periodontal repair.

Surgical treatment of periodontal defects typically involves flap surgery, bone grafting, and a higher risk of tissue damage or tooth loss [16]. In contrast, the application of CEM cement, following instrumentation, disinfection, and sealing of the endodontic space, enabled a less traumatic intervention that preserves tooth structure and promotes healing [17]. In addition, CEM cement simplified the treatment process, eliminating the need for complex surgical techniques and improving patient comfort and compliance. The nonsurgical, minimally invasive approach used in this study offered a notable advantage over traditional surgical methods with no complications.

This study highlights the role of bioactive materials in modern endodontics and periodontics, where bioactive cements are utilized not only for their sealing properties of furcation canals but also for their potential to encourage periodontal regeneration [18]. The antibacterial properties of CEM cement make it a valuable biomaterial for cases where periodontal health is compromised by endodontic-related furcation defects [19, 20]. The long-term follow-up period of an average of 26 months for the healed cases in this case series supports the favorable outcomes of CEM cement in the nonsurgical management of furcation defects. The minimal incidence of partial healing (Case #6) suggests that while CEM cement offers substantial benefits, further refinements in technique or supplementary periodontal treatments may be needed for certain cases to achieve complete resolution.

While this case series shows promising results, it has limitations, including a small sample size, lack of assessment of adjunctive systemic antibiotics, and inclusion limited to furcation defects of endodontic origin, which may not reflect outcomes in periodontal or combined lesions.

## 4. Conclusion

This case series demonstrates that CEM cement as a cervical seal offered effective sealing and regenerative potential, leading to favorable clinical outcomes, including the resolution of abscesses, reduction of probing depths, and restoration of tooth function. The findings of this study support the growing body of evidence that the calcium silicate cement-based materials, such as CEM cement, are a promising material for the nonsurgical management of furcation defects of endodontic origin. Further research with larger sample sizes and long-term follow-ups is needed to fully establish the clinical efficacy of this approach and expand its applicability in dental practice.

## Data Availability

The data that support the findings of this study are available from the corresponding author upon reasonable request.
